# Canine Enteric Coronaviruses: Emerging Viral Pathogens with Distinct Recombinant Spike Proteins

**DOI:** 10.3390/v6083363

**Published:** 2014-08-22

**Authors:** Beth N. Licitra, Gerald E. Duhamel, Gary R. Whittaker

**Affiliations:** 1 Department of Microbiology & Immunology, Cornell University College of Veterinary Medicine, Ithaca, NY 14853, USA; E-Mail: bnm4@cornell.edu; 2 Department of Biomedical Sciences, Cornell University College of Veterinary Medicine, Ithaca, NY 14853, USA; E-Mail: ged36@cornell.edu

**Keywords:** canine coronavirus, viral pathogenesis, spike protein, recombination

## Abstract

Canine enteric coronavirus (CCoV) is an alphacoronavirus infecting dogs that is closely related to enteric coronaviruses of cats and pigs. While CCoV has traditionally caused mild gastro-intestinal clinical signs, there are increasing reports of lethal CCoV infections in dogs, with evidence of both gastrointestinal and systemic viral dissemination. Consequently, CCoV is now considered to be an emerging infectious disease of dogs. In addition to the two known serotypes of CCoV, novel recombinant variants of CCoV have been found containing spike protein *N*-terminal domains (NTDs) that are closely related to those of feline and porcine strains. The increase in disease severity in dogs and the emergence of novel CCoVs can be attributed to the high level of recombination within the spike gene that can occur during infection by more than one CCoV type in the same host.

## 1. Introduction and Background

Canine enteric coronavirus (CCoV) is a common infection of dogs, particularly those housed in large groups such as kennels, shelters, and breeding facilities. CCoV belongs to the family *Coronaviridae*, order *Nidovirales*, and was first recognized as a pathogen of dogs following virus isolation of the prototype 1-71 virus in 1971 during an outbreak of gastroenteritis in military dogs [1]. Classically, CCoV was considered to cause only self-limiting enteritis with mild diarrheal disease [2]. However, CCoV has emerged as a significant pathogen in veterinary medicine, and is increasingly found to be an important cause of disease. The viral spike protein binds the host cell receptor and triggers fusion of the viral and cellular membranes. As such, it is an important determinant of cell tropism and pathogenicity [3]. Based on the high level of naturally occurring recombinations and mutations among coronaviruses, especially within the spike gene, there is the likelihood of continued emergence of novel CCoVs with distinct pathogenic properties in the future.

## 2. CCoV Structure and Genotyping

Coronaviruses are enveloped, single-stranded, positive-sense RNA viruses that infect humans and a wide variety of animal species [3,4]. Coronavirus particles are enveloped and are composed of four major structural proteins; spike (S), envelope (E), membrane (M), and nucleocapsid (N) [5]. The association of the *N* protein with the genomic RNA forms the helical nucleocapsid that is surrounded by an icosahedral structure composed of the viral M protein. The virus enters cells upon receptor binding and membrane fusion (mediated via the viral S protein) [6], and, like many other positive-sense RNA viruses, replicates within the cytoplasm in association with a complex membranous network called a viral factory [7]. Coronaviruses employ a unique mechanism of replication. The (+) strand RNA viral genome is transcribed into a full-length (−) strand RNA genomic template and (−) strand subgenomic templates for mRNA synthesis. RNA recombination is believed to occur during this process of (−) strand RNA synthesis. Subsequently, the (−) strand templates are transcribed to form the (+) strand RNA genomes and (+) strand nested subgenomic mRNAs [8]. While 3'-exonuclease activity allows some degree of proofreading activity [9], the coronavirus genome, as with all RNA viruses, is highly prone to mutation during replication. During virus assembly, the virion buds into the endoplasmic reticulum-Golgi intermediate compartment (ERGIC) where it enters the secretory pathway of the cell, allowing maturation and processing of the heavily glycosylated viral spike protein. The virus exits the cell via exocytosis. Research on coronaviruses has greatly increased since 2003 due to the emergence of severe acute respiratory syndrome (SARS) caused by a zoonotic transmission of an animal coronavirus into the human population [10].

**Figure 1:**
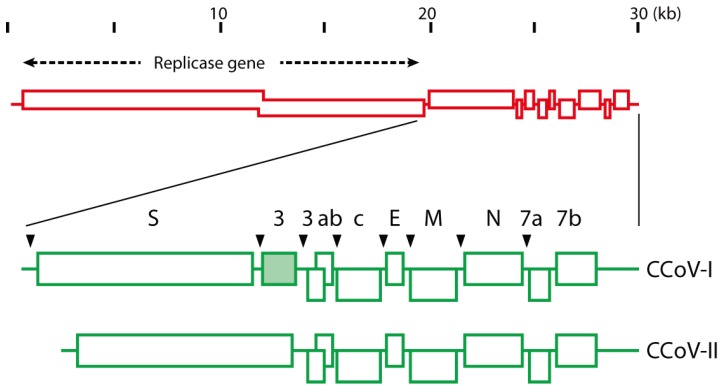
Image representation of the genomes of CCoV-1 and CCoV-II. Adapted from Lorusso *et al.* [22].

Coronaviruses are phylogenetically divided into several genera, termed alpha, beta, and gamma [11]. The existence of a fourth genus –deltacoronavirus– has also been suggested [12]. Classification of coronaviruses into different genera is based in part on the presence or absence of small open-reading frames situated downstream of the genes encoding the main structural proteins. There are two known types of canine coronaviruses: CCoV is a member of the alphacoronavirus genus [11], and the more recently identified canine respiratory coronavirus (CRCoV) is a member of the betacoronavirus genus [13]. CCoV is closely related to transmissible gastroenteritis virus (TGEV) of pigs, ferret coronavirus and feline coronavirus (FCoV) [11]; whereas CRCoV is more closely related to bovine coronavirus [14]. All enteric CCoVs (along with the related viruses of cats, pigs, and ferrets) are given the same strain designation (*Alphacoronavirus-1*) from a taxonomic perspective. However, there are two distinct serotypes of CCoV: type I and type II [15,16]. CCoV-I and CCoV-II, though closely related to each other, have markedly different spike proteins [17]—A situation analogous to that in cats, where feline coronaviruses (FCoV) also exist as two equivalent serotypes (type I and type II), based on antigenically distinct spike proteins [18]. It has been proposed that type I CCoVs and FCoVs evolved from a common ancestral virus, and that the canine and feline type II lineages arose from multiple recombination events with an unidentified genetic source [19]. A summary of the genome organization of CCoV-I and CCoV-II is shown in Figure 1. The group of CCoV-I viruses was identified initially based on analysis of CCoVs with a variant M gene [20]—with Elmo/02 as the prototype strain [20,21].

More extensive genetic characterization led to such variant viruses being termed “FCoV-like” CCoVs, based on their similarity to the commonly circulating FCoV strains (FCoV-I) [17]. Like other CCoV’s, these variant viruses can be sequenced from the feces of young dogs with diarrheal disease. Subsequently, these viruses were designated CCoV-I to discriminate them from previously identified viruses such as 1-71 (which were classified as CCoV-II). In addition to the genomic differences identified in earlier work, it is now known that the major differences between viruses belonging to CCoV-I and CCoV-II are primarily found within the spike protein, and account for the distinct serological properties of these two viruses. Notably, CCoV-I has a unique feature for coronaviruses, the presence of an intact open reading frame (ORF) 3 downstream of the gene encoding the spike protein, which encodes a glycoprotein of unknown function [19].

CCoV-I isolates are not culturable in cell culture systems, which has severely hampered the study of these viruses. *In vivo*, CCoV-I viruses are thought to co-circulate extensively with CCoV-II viruses, often occurring as co-infections [22,23,24,25,26,27,28]. This extensive co-circulation, combined with the capacity for recombination inherent in coronavirus replication, suggests that natural selection of novel recombinant viruses is not uncommon. Such a situation has been proposed for the divergent A76 CCoV [29] (isolated in 1976), as well as other similar viruses [30] that have CCoV-1/CCoV-II recombinant spike proteins. This indicates that CCoV-I-like viruses have been in circulation in dogs for a considerable time prior to the identification of Elmo/02.

The first identified CCoV strain 1-71, is now categorized as a type II virus (CCoV-II). Further classification of CCoV-IIs into distinct subtypes (CCoV-IIa, CCoV-IIb) has been proposed, based on the sequence of the first 300 amino acids of the spike protein, a region known as the *N*-terminal domain (NTD). The NTD is an important determinant of intestinal tropism in closely related TGEV [31,32]. The CCoV IIa and IIb classifications are not officially accepted within CCoV taxonomy, but are widely cited in the literature. CCoV-IIa viruses have a NTD consistent with the prototype CCoV. These viruses exist in two biotypes that differ in pathogenicity and tissue tropism. The “classical” CCoV-IIa biotype is restricted to the small intestine, where it causes enteritis. In contrast, the emergent “pantropic” CCoV-IIa biotype can spread systemically, causing leukopenia [33,34,35]. The viruses CB/05 and 450/07 have been studied extensively as prototype pantropic CCoVs [24,33,34,36,37]. The second variant, CCoV-IIb, is genetically distinct from CCoV-IIa, with the CCoV-IIb spike gene having a TGEV-like NTD [38,39]. Like TGEV, CCoV-IIb causes enteritis in neonatal animals. CCoV-IIb RNA has been detected by PCR assays in various organs outside of the intestinal tract, primarily in dogs that are co-infected with canine parvovirus [25,38] but also in dogs with uncertain disease status [34]. Isolate 341/05 has been proposed as a prototype CCoV-IIb [38].

**Figure 2:**
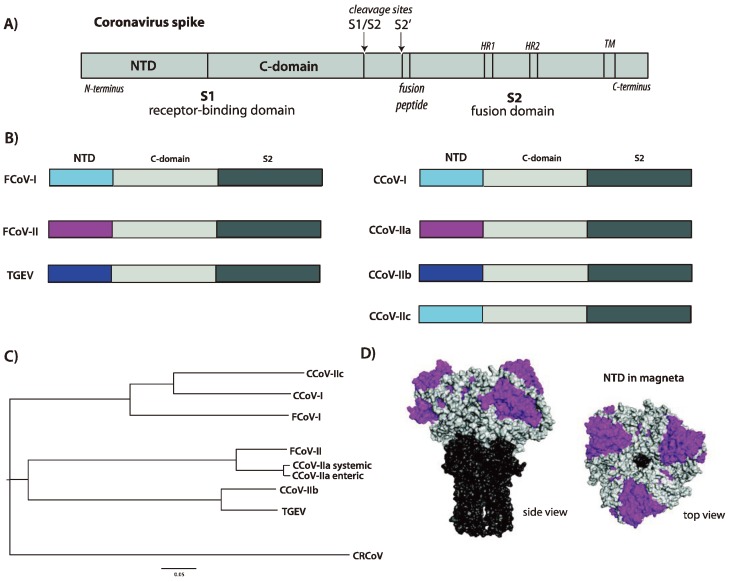
(**A**) Image representation of a coronavirus spike protein. Key features are identified, including the *N*-terminal domain (NTD) and C-domain within the S1 receptor-binding domain, and the fusion peptide, two heptad repeats (HR1 and HR2) and transmembrane domain (TM) in the S2 fusion domain. The two cleavage sites for protease activation (S1/S2 and S2’) are shown; (**B**) Image representation of the different domains present within the spike proteins of alpha-coronaviruses of cats (FCoV), dogs (CCoV) and pigs (TGEV). The different NTDs are color-coded to indicate homology and proposed recombination events across the species, with the remainder of S1 depicted in light gray and S2 in dark gray; (**C**) Phylogenetic tree of the NTDs present within the spike proteins of representative coronaviruses of cats (FCoV), dogs (CCoV) and pigs (TGEV) (The following virus sequences were used for alignment. NTD = aa 1-279 (based on CCoV-A76). FCoV-I = Black (BAC05493.1); FCoV-II = WSU-79-1146 (AGZ84516.1); CCoV-IIa = CCoV CB/05 (AAZ91437.1); CCoV-IIb = CCoV 341/05 (ACJ63231.1); CCoV-I = CCoV Elmo/02 (AAP72149.1); CCoV-IIc = CCoV-A76 (AEQ61968.1); CRCoV = CRCoV4182 (ABG78748.1); TGEV (CAB91145.1)). The tree was created using ClustalX [45] and FigTree software [46]; (**D**) Structural model of a coronavirus spike protein based on PDB file 1T7G [47], in surface rendering. The NTD is colored magenta, the remainder of S1 is light gray and the S2 domain is colored dark gray. Two views of the trimeric spike are shown, a side view and a top view.

Based on the finding of a novel NTD in A76-like viruses, we propose the creation of a new subgroup, CCoV-IIc, which consists of CCoV type II viruses with a CCoV-I- or FCoV-I-like *N*-terminal domain. These viruses have been reported in both the United States and Sweden [29,30]. Other viruses with similar biological properties may have also been identified in the past, including an atypical CCoV from a breeding facility in 1997 [40]. As CCoV-A76 has been isolated and characterized [29], this serves as a convenient prototype CCoV-IIc. An image of the various spike protein domains for CCoVs, and a summary of NTD phylogeny, is shown in Figure 2.

CCoV-II viruses (such as 1-71) typically grow readily in cell culture, with A-72 cells (canine tumor fibroblast cells derived from unknown tissue type) widely used for virus propagation. 1-71 also grows well in a variety of feline cell lines (e.g., CRFK), but, interestingly, not in many other canine cell lines [29].

## 3. Functional Aspects of the CCoV Spike Protein

### 3.1. Receptor Binding and Host Tropism

The coronavirus spike protein is a major antigenic determinant and is also responsible for host cell receptor binding and viral entry [41]. Aminopeptidase *N* (APN) has been shown to act as a common receptor for many alphacoronaviruses, including FCoV and TGEV [42]. Although each virus would be assumed to utilize a species-specific homolog in its respective host during infection, the feline homologue (fAPN) has been shown to act as a common receptor for type II FCoV, type II CCoV and TGEV [43]. This property is unlike most other coronaviruses, which have highly species-specific receptors. Such broad receptor-binding ability has likely played a role in the zoonotic transfer and genetic recombination events that have defined the evolution of animal alphacoronaviruses. Feline and human APN are also receptors for the human alphacoronavirus HCoV-229E, but not for another human alphacoronavirus, NL63 [43,44]. Presumably, the canine APN acts as the *in vivo* receptor for all CCoV-IIs, as shown for 1-71 [29]. In the case of the CCoV-IIc A76, the presence of a divergent APN-binding domain within the spike glycoprotein, the “*C*-domain”, has resulted in the ability to use cAPN but not fAPN as a receptor. The relative lack of glycosylation on fAPN compared to cAPN may account for these differences [29].

The receptor determinants for the serotype I FCoV/CCoV group are much less certain. While there is some evidence for fAPN as an FCoV type I receptor [43], other studies have concluded that there is a distinct receptor for FCoV type I [48,49]. Overall, receptor determinants for CCoV type I viruses remain essentially unknown.

In addition to a specific proteinaceous receptor, there are indications that lectin-based interactions via sugar moieties, on either the virus or the host, may play a role in the receptor-binding complex for FCoV types I and II and TGEV [31,32,50,51,52] and the same situation may also apply to CCoVs. Many coronaviruses also bind sialic acid, which can be an important determinant of tissue tropism and pathogenesis, but this has not been investigated for FCoVs or CCoVs.

### 3.2. Activation by Proteolytic Cleavage

**Figure 3:**
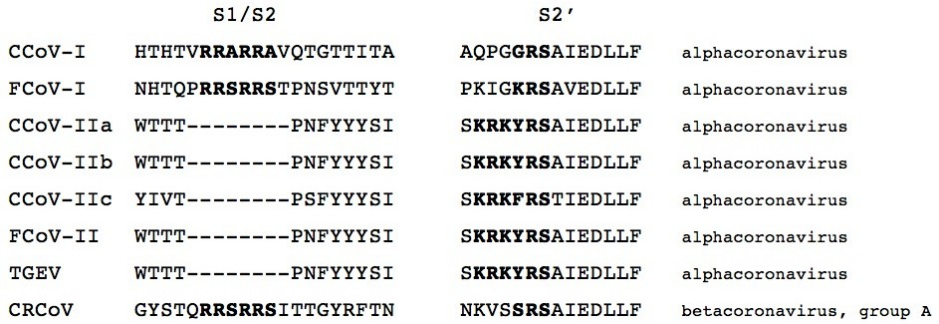
Multiple sequence alignment of the spike proteins of representative coronaviruses of cats (FCoV) dogs (CCoV and CRCoV) and pigs (TGEV) (The following virus sequences were used for alignment. FCoV-I = RM (ACT10854); FCoV-II = WSU-79-1683 (AFH58021.1); CCoV-IIa = CCoV CB/05 (AAZ91437.1); CCoV-IIb = CCoV 341/05 (ACJ63231.1); CCoV-I = CCoV Elmo/02 (AAP72149.1); CCoV-IIc = CCoV-A76 (AEQ61968.1); CRCoV = CRCoV4182 (ABG78748.1); TGEV (CAB91145.1).), in the region of the two activation sites (S1/S2 and S2’). The alignment was created using ClustalX [45]. The expected cleavage motif is in bold.

The coronavirus spike protein is activated by proteolytic cleavage at one or two sites (defined as S1/S2 and S2’) [8]. In other virus systems, notably influenza viruses and Newcastle disease virus, modification of these sites is an important pathogenicity determinant [53]. Depending on the individual coronavirus, cleavage at S1/S2 may or may not occur [6]. S2’ cleavage is likely a more universal requirement, and is linked directly to exposure of the viral fusion peptide [54,55,56]. Cleavage at the two sites can occur via the action of a wide range of proteases, e.g., trypsin or trypsin-like proteases, cathepsins, elastase, or furin [57]. The FCoV/CCoV type I and II lineages present an interesting and distinct difference in spike protein cleavage-activation. The type I lineage shows the presence of two distinct protease cleavage motifs (R-R-S/A-R-R-S/A at S1/S2 and G/K-R-S at S2’), whereas the type II lineage is missing an obvious motif at S1/S2 and has a single distinctive R-K-R-Y/F-R-S cleavage motif at S2’. This implies that the viruses in these two lineages have quite distinct means of cleavage activation. A multiple sequence alignment summarizing these sites is shown in Figure 3. While mutations in the FCoV cleavage site have been linked to a change of cleavability by the protease furin (which cleaves readily at R-R-S/A-R-R-S/A motifs) and correlate with gain of macrophage tropism and the development of FIP in cats [58], there is currently no evidence for cleavage site mutations in highly pathogenic CCoV infections.

## 4. CCoV Pathogenesis and Clinical Presentation

CCoV is transmitted by the fecal-oral route; however, it is unclear whether transmission by other routes including aerosols can occur. After ingestion, CCoV typically infects and replicates within the cytoplasm of mature epithelial cells along the sides and the tip of intestinal villi, while sparing the epithelium that lines the intestinal crypts (Figure 3). Infected villous enterocytes undergo degeneration characterized by shortening, distortion and loss of microvilli of the brush border, leading to sloughing of necrotic cells into the lumen. The loss of mature villous enterocytes causes atrophy of intestinal villi, which become attenuated in an attempt to maintain the integrity of the intestinal barrier. In response to the loss of villous enterocytes, there is an increase in mitotic activity of the crypt epithelium and a net expansion of the pool of immature enterocytes. Together, these changes in the small intestinal morphology translate into a loss of normal digestive and absorptive functions and the clinical signs of diarrhea and dehydration in affected dogs [59].

CCoV is generally thought of as a mild, but highly contagious, enteritis of young dogs, most often under 12 weeks of age [2,60,61]. In some cases, CCoV infection can be fatal, particularly in puppies co-infected with other pathogens such as canine parvovirus [62,63]. Therefore, the range of clinical signs from loose stools to severe watery diarrhea with high morbidity and variable mortality is mainly determined by the age at the onset of infection, the level and type of pathogen exposure, and the degree of maternal transfer of immunity.

In recent years, an increasing number of reports of infections by highly virulent CCoVs have also been documented in puppies without apparent coinfections [24,25,30,36,37,64,65,66,67,68,69]. In the case of the “pantropic” CCoV-IIa viruses, infection results in a fatal multisystemic illness, with various clinical signs similar to canine parvovirus infection, and including high fever, hemorrhagic gastroenteritis, neurological signs, and lymphopenia. The most consistent clinical sign of pantropic CCoV-IIa is leukopenia [33,34,35]. Most studies of pantropic CCoV-IIa report the presence of viral RNA in various tissues, including lungs, lymph nodes, liver, spleen, kidneys, urinary bladder and brain. Evidence for replication of these pantropic viruses outside of the gastrointestinal tract is based on demonstration of CCoV antigen by immunohistochemical staining of lung tissue taken from a single dog [24,37], and reports of virus isolation from various visceral organs [24,33,34]. Viral isolation is often unsuccessful, even from tissues with the highest viral RNA levels [25,34]. The genetic markers for pantropic CCoV-IIa’s are currently unknown. TGEV-like CCoV-IIb has also been associated with systemic spread, but only in cases of co-infection with canine parvovirus. Some features of systemic infection that are similar to severe acute respiratory syndrome (SARS) in humans and feline infectious peritonitis (FIP) in cats, have been documented for systemic fatal CCoV-II infections [37], including pulmonary alveolar damage, fibrinous exudation and macrophage involvement. In spite of similarities between these viruses and frequent sub-clinical infections, it is clear that systemic and lethal FIP is a much more common outcome for FCoV infection of cats, compared to CCoV infection of dogs.

## 5. Recombination and Emergence of CCoV Variants with Altered Pathogenicity

It is clear that coronaviruses have high potential for emergence of novel variants with altered tropism and pathogenesis. In large part this can be explained by several factors: a combination of recombination and mutation within the viral genome and that many coronaviruses exist as quasi-species, the availability of natural reservoirs for the virus and the “modular” nature of the viral spike protein [70]. Recent studies have highlighted the extraordinary complexity of canine coronavirus genomics. The increased likelihood of high-density housing for dogs increases the viral load of coronavirus in the population, and increases the likelihood of novel viruses emerging within dogs. It is now well-established that the feline coronavirus FIPV WSU-79-1146 arose following recombination with a canine coronavirus [71], and other recombinant canine-feline viruses have been identified [72]. Increased co-housing of dogs with other species, particularly cats, also increases the chance of novel recombinant coronaviruses emerging across species.

## 6. Laboratory Diagnosis

**Figure 4:**
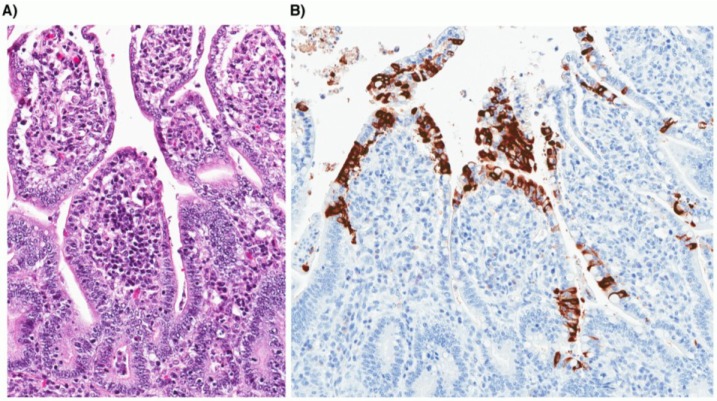
Photomicrographs of small intestine taken from a two-week-old puppy with typical lesions of CCoV infection. (**A**) Severe atrophy of intestinal villi with attenuated low cuboidal to squamous enterocytes (hematoxylin and eosin stain; 20× original magnification). (**B**) Coronavirus antigen is present within the cytoplasm of infected villous enterocytes (immunohistochemistry; 20× original magnification).

Definitive diagnosis of CCoV-induced disease is currently difficult; thus, it is not commonly done or available. The virus can be identified by visualization of viral particles in stool specimens following negative staining and examination by transmission electron microscopy; however, this is not a routinely available diagnostic tool. Virus isolation can be achieved for CCoV-II, but not for CCoV-I viruses, and again, this is not commonly available. The definitive test is post-mortem identification of viral antigen by immunofluorescence or immunohistochemical staining of tissue sections (see Figure 4). The most useful ante-mortem tests are RT-PCR-based, which are highly sensitive assays [22,73,74]. A common PCR test can reliably detect alphacoronaviruses [75], and more specific PCR tests can be employed for further characterization into specific serotypes or genotypes; however, because of the highly variable nature of CCoV genomes, novel variants may be missed with this approach. Serological tests are of limited use since they can only confirm exposure to CCoV and cannot currently discriminate between infecting CCoV serotypes or genotypes.

## 7. Vaccination and Treatment

A variety of inactivated and modified-live virus vaccines are commercially available and designed to prevent infection with CCoV [59]. Current vaccines are safe, but provide only incomplete protection—in that they reduce, but do not eliminate, CCoV replication in the intestinal tract [40,76]. As these vaccines are likely based on the classical CCoV-IIa viruses, protection against CCoV-1 strains with these vaccines is unlikely, and protection against the variant type II strains is uncertain. Treatment of CCoV-induced gastroenteritis is mainly by supportive care, including good maintenance of fluid and electrolytes. There are no available anti-viral drugs for treatment of CCoV infections.
